# Louseborne Relapsing Fever among East African Refugees, Italy, 2015

**DOI:** 10.3201/eid2202.151768

**Published:** 2016-02

**Authors:** Anna Lucchini, Filippo Lipani, Cecilia Costa, Mariaelisabetta Scarvaglieri, Rosanna Balbiano, Sinibaldo Carosella, Andrea Calcagno, Sabrina Audagnotto, Anna Maria Barbui, Silvia Brossa, Valeria Ghisetti, Ivano Dal Conte, Pietro Caramello, Giovanni Di Perri

**Affiliations:** Ospedale Amedeo di Savoia, Turin, Italy (A. Lucchini, F. Lipani, R. Balbiano, S. Carosella, S. Audagnotto, V. Ghisetti, I. Dal Conte, P. Caramello);; Università degli Studi di Torino, Turin (C. Costa, M. Scaravaglieri, A. Calcagno, S. Brossa, G. Di Perri);; Città della Salute, Turin (A.M. Barbui)

**Keywords:** Relapsing fever, Borrelia recurrentis, Italy, refugees, East Africa, lice, vector-borne infections, bacteria

## Abstract

During June 9–September 30, 2015, five cases of louseborne relapsing fever were identified in Turin, Italy. All 5 cases were in young refugees from Somalia, 2 of whom had lived in Italy since 2011. Our report seems to confirm the possibility of local transmission of louse-borne relapsing fever.

Louseborne relapsing fever (LRF) was once widely distributed in all geographic areas, including Europe and North America, occurring in association with poverty and overcrowding. In Europe, it virtually disappeared after World War I in parallel with improved living conditions that led to substantially decreased body lice infestations in humans (*1*). Currently, LRF is reported mostly from Ethiopia and surrounding countries, where it is endemic (*2*): in this region, it is an extremely common infection with substantial mortality. The causative agent is the spirochete bacterium *Borrelia recurrentis*. In nature, the only relevant vector is the body louse, which feeds only on humans; no other reservoir for this infection is known (*1*,*3*). The incubation period is 3–12 days. We report 5 cases of LRF in refugees to Italy from East Africa that occurred during 2015.

## The Cases

**Figure 1:**
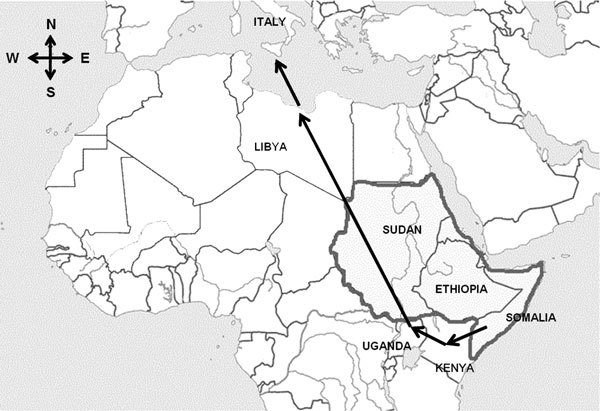
Route (arrows) followed by refugees from Somalia to Libya, where they boarded a boat to Sicily. Gray shading indicates *Borrelia recurrentis*–endemic countries.

All 5 patients were young men from Somalia (Table). Patients 1, 4, and 5 had recently arrived in Italy after traveling from Somalia through Kenya, Uganda, and Sudan to Libya, where they boarded a boat to Sicily (Figure 1). Patients 2 and 3 had resided in Italy since 2011, and both denied any travel outside Europe in the past 4 years. These 2 men lived in the same building in Turin, occupied by ≈600 refugees of different nationalities, most of them from Somalia. Patients 4 and 5 also reported a short stay in the same building.

**Table:** Characteristics of louseborne relapsing fever among East African refugees, Italy, 2015*

Characteristic	Patient 1	Patient 2	Patient 3	Patient 4	Patient 5
Time from arrival in Italy	3 d	4 y	3 y	10 d	10 d
Age, y	20	27	31	20	26
Date of admission	Jun 9	Jul 28	Sept 5	Oct 1	Sept 29
Date of symptom onset	Jun 7	Jul 24	Sept 1	Sept 27	Sept 26
Symptom					
Fever	Yes	Yes	Yes	Yes	Yes
Abdominal pain	No	No	Yes	Yes	No
Vomiting	Yes	Yes	Yes	Yes	No
Diarrhea	No	No	Yes	No	No
Headache	Yes	Yes	Yes	Yes	Yes
Myalgia	No	Yes	No	No	Yes
Other	None	Lumbar pain	None	Cough	Chest pain, itching
Laboratory test (reference)					
Platelets (>150,000/μL)	32,000	47,000	22,000	41,000	2,9000
Bilirubin (<1.2 mg/dL)	2.3	1.8	3.9	4.2	1.8
Liver function†	Normal	Normal	AST ×4; ALT ×3	AST ×3; ALT normal	Normal
Prothrombin time (>70%)	78%	66%	68%	100%	70%
CRP†	×16	×56	×95	×115	×59
Procalcitonin (<0.5)	11.36	NA	21	11.9	NA
QTc (<440 msec)	428	361	423	391	414
PCR for *Borrelia recurrentis*	Positive	Positive	Positive	Positive	Positive
Treatment	Doxycicline	Doxycicline	Doxycicline	Doxicicline + ceftriaxone	Doxycicline, switched to ceftriaxone
Jarisch-Herxheimer reaction	Yes, mild	Not observed	Not observed	Not observed	Yes, moderate

*ALT, alanine aminotransferase; AST, aspartate aminotransferase; CRP, C-reactive protein; NA, not available; QTc, corrected QT interval. †Indicated as normal (within reference value) or × upper reference value.

All patients sought care at one of the city emergency departments (EDs), reporting a 2–4-day history of fever with chills and headache. Other common symptoms included vomiting, myalgia, and abdominal pain. One patient had diarrhea.

Routine blood exams, performed on all 5 men, showed marked thrombocytopenia (22,000–48,000/μL) and elevated C-reactive protein (values 16–115 times the upper reference value). Procalcitonin, measured in 3 patients, was markedly increased (11.4–21 ng/mL [reference <0.5 ng/mL]). Liver function tests and bilirubin were either normal or slightly elevated.

**Figure 2:**
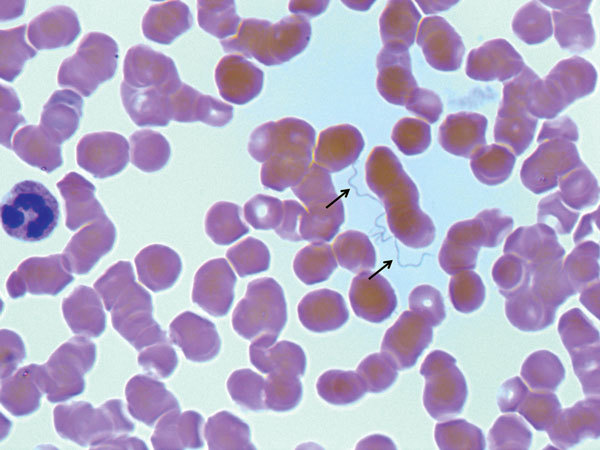
Giemsa-stained thin blood smear showing spirochetes (arrows) in a 27-year-old male refugee from Somalia (patient 2) with louseborne relapsing fever, Italy, 2015. Original magnification ×1,000.

All patients were transferred to the Infectious Disease Hospital in Torino for further assessment. Giemsa-stained thin and thick blood smears were negative for malaria parasites but showed spirochetes (Figure 2). LRF was suspected, and the patients were treated with either doxycycline alone or doxycycline plus ceftriaxone. Patients 1 and 5 showed an acute febrile reaction after the first antimicrobial dose: symptoms were compatible with a Jarisch-Herxheimer reaction (JHR).

No lice were found on the patients or on their clothes; patient 5 had skin lesions caused by scratching. None of the patients had rash or bleeding as described in the literature (*3*). Hepatomegaly was not observed; only patient 4 showed an enlarged spleen at ultrasound. Tachycardia was common, but alterations of cardiac function were not observed (no murmurs, corrected QT interval in normal range). A low systolic blood pressure (<90 mm Hg) was observed only in patient 4.

Bacterial DNA was extracted from 200 μL of blood from each of the 5 patients by using the QIAmp Mini Kit (QIAGEN, Hilden, Germany) and was detected amplifying the 16S rRNA. Nucleotide sequences of PCR products were determined. Sequences were identified by BLAST (http://blast.ncbi.nlm.nih.gov/Blast.cgi). *B. recurrentis* was identified in all patients, showing 100% identity with sequences of *B. recurrentis* reference strain A1 (GenBank accession nos. NR074866 and CP000993).

Italy has recently received large numbers of refugees from East Africa, particularly from Somalia. These refugees come from and travel through countries where *B. recurrentis* is endemic; along the way, they are often sheltered in crowded conditions with very poor hygienic facilities. Two of the patients reported here indicated that, while staying in Libya, they were held with many other persons in a close environment, and all refugees housed together reported severe itching.

Many of these refugees enter Italy through Sicily, from where they are sent to reception centers throughout the country. Some of these reception centers have grown to substantial size and now house a more stable population, with continuous input of new arrivals. In these conditions, local transmission can occur with a possible risk for epidemics: 2 of the 5 patients reported here were long-term residents in Italy, and they denied recent travel to Africa, so they probably acquired the infection while being housed in the same facilities as the newly arrived refugees. Although it is possible that they denied recent travel for fear of legal consequences, they are unlikely to have had the opportunity to travel out of Europe for economic reasons.

We did not find any louse on the body or in the clothing of these patients. However, we identified *B. recurrentis* by gene sequencing, and the dynamic of transmission we postulate in our cases fits more closely the model of louse transmission (overcrowding, poor hygienic conditions, migration) than that of tick transmission (*1*,*3*,*4*). Lice are relatively short-lived and remain infected throughout their lives but cannot transmit borreliae to their progeny. They do not inject borreliae directly while feeding (infection takes place when they are crushed on skin) (*1*): infected lice can survive on uninfected persons but, when moved to other persons, give rise to infection. Thus, identifying a direct chain of transmission is often challenging. The presence of lice has been reported as a growing problem in western Europe homeless persons with no history of travel, threatening an alarming scenario (*4*,*5*).

All 5 patients reported here sought care at one of the EDs in Turn for fever associated with other nonspecific symptoms; all 5 diagnoses were made as occasional observations of spirochetes on thin and thick blood smears conducted to search for malaria parasites. These 5 patients might represent a minority of persons who are actually affected by relapsing fever. In fact, in many instances, persons seen at an ED for fever, especially persons without a history of recent travel to malaria-endemic countries, would not be investigated for malaria and would probably be treated empirically with antimicrobial drugs, leading to resolution of symptoms without further investigations.

Two of the patients we report had symptoms suggestive of a JHR. JHR is an acute, febrile reaction, potentially fatal, occurring shortly after starting antimicrobial drugs, probably attributable to the release of cytokines associated with clearance of borreliae from blood (*2*,*6*). A higher frequency of JHR has been reported in the literature, up to 80% (*6*,*7*), with some differences depending on the antimicrobial drug used (*7*,*8*). However, most observations were performed in LRF-endemic countries. These patients were all hospitalized and were receiving supportive treatment (intravenous rehydration and paracetamol, either intravenously or orally, to control headache, myalgia, and abdominal pain), which might have played a role in avoiding JHRs or in reducing its symptoms. Several studies have been conducted on possible preventive options to avoid the arousal of JHR, but results have not been consistent (*9*). Some clue of a possible action of paracetamol on the development of JHR has been reported (*8*).

## Conclusions

In summary, we identified *B. recurrentis* infection in 5 patients in Italy who were refugees from East Africa; 2 of these patients had not traveled outside Italy for several years. Beginning in July 2015, several reports from countries in Europe have described relapsing fever in refugees from East Africa (*10*–*13*). In some of these cases, transmission might have occurred during transit through Italy (*12*). Our findings confirm the possibility of local transmission of LRF caused by *B. recurrentis*.
